# A Cell Membrane-Level Approach to Cicatricial Alopecia Management: Is Caveolin-1 a Viable Therapeutic Target in Frontal Fibrosing Alopecia?

**DOI:** 10.3390/biomedicines9050572

**Published:** 2021-05-19

**Authors:** Ivan Jozic, Jérémy Chéret, Beatriz Abdo Abujamra, Mariya Miteva, Jennifer Gherardini, Ralf Paus

**Affiliations:** 1 Dr. Phillip Frost Department of Dermatology and Cutaneous Surgery, University of Miami Miller School of Medicine, Miami, FL 33186, USA; jpc219@med.miami.edu (J.C.); bxa520@med.miami.edu (B.A.A.); mmiteva@med.miami.edu (M.M.); Jxg2067@med.miami.edu (J.G.); Rxp803@med.miami.edu (R.P.); 2 Monasterium Laboratory, Skin and Hair Research Solutions GmbH, 48149 Münster, Germany; 3 Centre for Dermatology Research, University of Manchester and NIHR Manchester Biomedical Research Centre, Manchester M13 9PT, UK

**Keywords:** caveolin-1, frontal fibrosing alopecia, cicatricial alopecia, immune privilege, hair follicle, cyclodextrin, statin

## Abstract

Irreversible destruction of the hair follicle (HF) in primary cicatricial alopecia and its most common variant, frontal fibrosing alopecia (FFA), results from apoptosis and pathological epithelial-mesenchymal transition (EMT) of epithelial HF stem cells (eHFSCs), in conjunction with the collapse of bulge immune privilege (IP) and interferon-gamma-mediated chronic inflammation. The scaffolding protein caveolin-1 (Cav1) is a key component of specialized cell membrane microdomains (caveolae) that regulates multiple signaling events, and even though Cav1 is most prominently expressed in the bulge area of human scalp HFs, it has not been investigated in any cicatricial alopecia context. Interestingly, in mice, Cav1 is involved in the regulation of (1) key HF IP guardians (TGF-β and α-MSH signaling), (2) IP collapse inducers/markers (IFNγ, substance P and MICA), and (3) EMT. Therefore, we hypothesize that Cav1 may be an unrecognized, important player in the pathobiology of cicatricial alopecias, and particularly, in FFA, which is currently considered as the most common type of primary lymphocytic scarring alopecia in the world. We envision that localized therapeutic inhibition of Cav1 in management of FFA (by cholesterol depleting agents, i.e., cyclodextrins/statins), could inhibit and potentially reverse bulge IP collapse and pathological EMT. Moreover, manipulation of HF Cav1 expression/localization would not only be relevant for management of cicatricial alopecia, but FFA could also serve as a model disease for elucidating the role of Cav1 in other stem cell- and/or IP collapse-related pathologies.

## 1. Background

Primary lymphocytic cicatricial alopecias (PLCA) are characterized by progressive, permanent hair loss for which there is currently no cure [1,2,3,4]. PLCA result from irreversible damage to the epithelial stem cells (eSCs) of the hair follicle (HF) due to (1) eHFSC apoptosis, (2) pathological epithelial-mesenchymal transition (EMT), (3) collapse of bulge immune privilege (IP) and (4) interferon-gamma-dominated Th1-type chronic inflammatory response [5,6,7,8]. Given the associated psychological distress [9], loss of normal skin function, and secondary morbidity of PLCAs [1,10,11], it is particularly unfortunate that currently available therapeutic options (such as topical corticosteroids, retinoids, tacrolimus, finasteride, minoxidil, pioglitazone) are at best unsatisfactory [10,12,13,14,15,16,17]. Therefore, the field is challenged to rapidly develop more effective, well-tolerated, and ideally topically applicable PLCA therapies [18].

Frontal fibrosing alopecia (FFA) is now the most frequent form of PLCA [19], which exemplifies typical eHFSC pathology, including bulge IP collapse and EMT, and has been advocated as a model eSC disease in which inflammation-associated, pathological eSC apoptosis and EMT can be exemplarily studied [6]. Despite identification of the aforementioned key characteristics, etiology and pathogenesis of FFA remain unclear. Moreover, this PLCA has recently seen an unexplained, almost “epidemic” increase in both prevalence and incidence [20,21,22,23]. To explain this, several hypotheses about environmental causes are considered plausible, including prolonged use of hormonal contraceptives [24,25] as well as the increased or excessive use of leave-on facial skin care/antiaging products and sunscreens [26] (particularly chemical sunscreens [27], and personal care products such as cleansers, cosmetics, creams, lotions, hair care products, etc). The association of leave-on cosmetics such as sunscreens with FFA is increasingly being proposed in the field [6,26,28]. For example, a higher frequency of sunscreen usage and positive patch tests to sunscreen ingredients in women with FFA has been reported by Moreno-Arrones et al. and Prasad et al. [27,29]. In addition, Kidambi et al. [28] found a significant relation between FFA and leave-on facial products, such as moisturizing creams and sunscreens in men. In general, topical application of creams/lotions tends to penetrate the hair canal until the level of the bulge, which would explain why the intensive and increasingly widespread use of these leave-on cosmetics would affect primarily the bulge and might weaken its relative IP, resulting in inflammation, which in turn could facilitate pathological EMT of bulge eSCs—two key mechanisms in the primary pathobiology of FFA and Lichen planopilaris (LPP) [6,8]. Though this association has been disputed [30,31], it is interesting to note that many of these products contain allergens (gallates, linalool, fragrance mix), which could be associated with the disease activity in patients with FFA and LPP: after at least 3 months of allergen avoidance, 70% of patients have decreased scalp erythema on examination [29]. Most interestingly, some of these allergens are also odorants, which may be important since selected odorants can indeed regulate human HF growth by stimulating defined olfactory receptors [32,33].

There has also been a proposed association between beta-blockers and lichen planus, lichenoid drug reactions and LPP [34], while Clayton et al. suggested an association between mucosal lichen planus and beta-blockers [35]. In a different series of 60 patients with FFA, 11% were on beta-blockers, which is a similar proportion of patients compared to Clayton et al.’s study, but a higher proportion were taking Non-steroidal anti-inflammatory drugs (NSAIDs) (11%, or 28% if aspirin included, vs. 4%, *p* < 0.001). Lastly, it has been suggested that beta-blockers indeed can cause (in a minority of patients) telogens effluvium due to premature catagen induction in anagen HFs and that beta blockers profoundly regulate HF cycling (in mice), which also prominently express beta2 adrenergic receptors in their stem cell zone (bulge) [36].

Histologically, lesional HFs in FFA display a lichenoid inflammatory cell infiltrate and perifollicular fibrosis with eventual replacement of the entire HF with fibrous tissue (fibrotic tracts) [6]. In contrast to the inflammation seen in alopecia areata (the most common autoimmune hair disease), where massive inflammation can attack the proximal bulb (reversibly without destroying the HF) of pigmented HF [37], in FFA, even minute infiltrates around the HF bulge are sufficient enough to damage the HF irreversibly. The central pathogenic process in FFA is an inflammation-induced permanent loss of eSCs in the HF bulge region, which are vital for HF cycling and regeneration [6,38,39,40,41]. This is preceded by loss of bulge IP, increased apoptosis of eHFSCs and pathological EMT of these bulge stem cells [6,7,8]. To which extent mitochondrial defects contribute to this bulge pathology is currently under investigation [33].

Understanding the mechanisms by which a relative IP niche in the bulge [42] is created, as well as those by which these protective mechanisms fail (bulge IP collapse) [7,42], constitute key problems in general human SC biology. Given that the specific niche into which eSCs are embedded (including its extracellular matrix environment and neural signals) dictates SC behavior and survival [6,43,44,45], FFA offers a superb, accessible, and clinically relevant model disease for exemplarily dissecting the pathological consequences of failure to preserve individual constituents and functions of the eSC niche.

**Figure 1:**
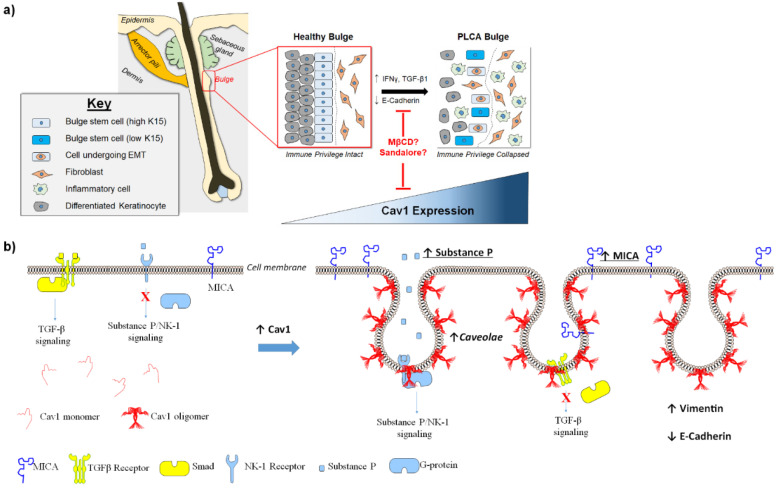
Proposed mechanism for the role of Caveolin-1 in development of FFA. (**a**) Inflammation-induced downregulation of E-cadherin along with excessive IFNγ and EGF signaling promote pathological EMT in bulge epithelial cells of human scalp HFs. (**b**) Upregulation of caveolin-1 (Cav1) expression allows for monomeric Cav1 to oligomerize at the cell membrane and induce formation of caveolae. Cav1 antagonizes TGF-β signaling by sequestering TGF-β receptors and preventing phosphorylation and signaling through Smad2, inhibiting its association with Smad4, as well as subsequent Smad2 nuclearization and TGFβ-mediated transcriptional activation. Conversely, upregulation of Cav1 leads to increased Substance P levels and promotes localization of its receptor NK-1 to caveolae where NK-1 interacts with downstream G-protein, resulting in sustained Substance P signaling through the NK-1 receptor. Lastly, upregulation of Cav1 has been associated with upregulation of MICA and Vimentin, as well as a downregulation of E-cadherin. Therefore, upregulation of Cav1 orchestrates development of environment permissive to eHFSC IP collapse by (1) inhibiting guardians of IP (TGF-β signaling), (2) promoting suppressors of IP (Substance P and MICA), and (3) promoting EMT (upregulating vimentin and downregulating E-cadherin).

Epithelial-to-mesenchymal transition (EMT) is a process by which epithelial cells lose polarity, cell-to-cell contact and acquire a mesenchymal phenotype. Interestingly, lesional FFA HFs show cells within the bulge epithelium that are abnormally positive for the EMT markers (Snail1, Snail2, Zeb1 and TWIST1) [46], mesenchymal markers (vimentin and fibronectin) and a cadherin switch (downregulation of the epithelial cell–cell adhesion marker E-cadherin [8] and upregulation of the N-cadherin) [47,48]. Since some K15 (Keratin 15, a bona fide marker of eHFSCs) positive cells also co-express vimentin [43], this suggests that some eHFSCs in FFA HFs undergo EMT [43] and contribute to the prominent fibrosis observed in FFA, which in addition to eHFSC apoptosis observed in FFA, functions to deplete the bulge stem cell niche even further. We have also demonstrated that a defined cocktail of four well-known EMT-promoting agents (i.e., IFN-γ, TGF-β1, EGF, and a E-cadherin-inhibiting peptide) [6,8] is sufficient enough to induce pathological EMT and reduce both expression of K15, as well as the number of K15+ cells in the bulge of healthy, human scalp HFs ex vivo [18], recapitulating exactly what is seen in lesional LPP and FFA HFs [6,7,8,18] (Figure 1a).

In this context, the plasma membrane of eHFSCs, namely its caveolae and their principal structural component, caveolin-1 (Cav1) [49,50], has not yet been considered. Cav1 compartmentalizes various signal transduction molecules and thus orchestrates numerous transmembrane signaling events involved in epithelial cell function including (but not limited to) endocytosis, lipid transport, signal transduction, inflammation, as well as cellular migration and proliferation (Figure 1b) (reviewed in [3]). Moreover, Cav1 has been shown to interact with and affect expression as well as activity of numerous inflammatory mediators (IL-1β, IL-2, IL-6, IL-12, TNF-α, etc.) [51], EMT (Vimentin, E-cadherin, Twist, fibronectin, etc.) [52], apoptotic markers (Bax, Bcl-2, caspase-3, caspase-9, cyclin D1, PARP, survivin, etc.) [53] and various signaling molecules (among others, Erk1/2, FAK, IGF-1, PI3K/Akt, Ras, Src, etc.) [49,54]. Although a few recent studies have begun to characterize expression and the potential functional role of Cav1 in skin epithelium [50,55,56,57,58,59,60], its role in human hair physiology remains entirely unknown. Here, we argue that Cav1 deserves special scrutiny and therapeutic targeting in future PLCA management, namely in the treatment of FFA.

## 2. Premises

We have previously shown that Cav1 localizes to the basal layer epidermal keratinocytes where it antagonizes keratinocyte migration and proliferation. Subsequently, spatiotemporal downregulation of Cav1 is required for appropriate cutaneous wound healing, and topical formulations which perturb Cav1 can accelerate wound closure using in vitro human skin organ cultures, human ex vivo wounds, as well as both murine and porcine in vivo wounds [55,56]. Given that eHFSCs critically participate in later stages of cutaneous wound healing (e.g., by producing progenitor cells that migrate out of the bulge into the epidermis to facilitate re-epithelialization [61,62,63]), and the fact that Cav1 downregulation is required for wound closure [55,56], the role of Cav1 might extend to epithelial HF progenitor cells and their emigration into the epidermis. If Cav1 is prominently expressed in the bulge itself, it might also serve more general functions as a physiologic regulator of eHFSCs proliferation/migration, similar to its function in basal layer epidermal keratinocytes [55,56].

**Figure 2:**
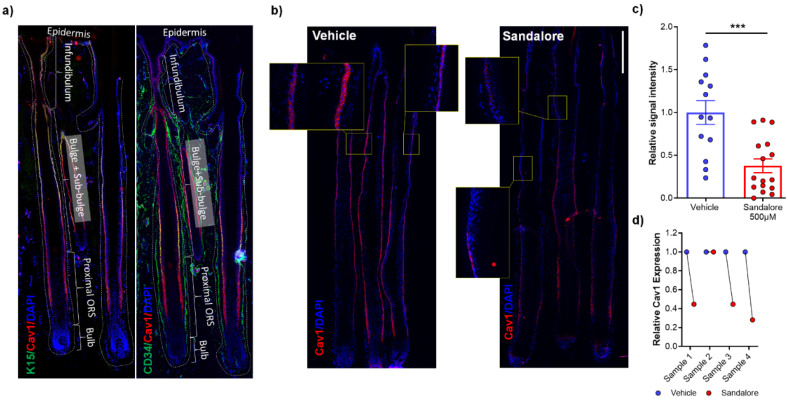
Caveolin-1 colocalizes with markers of eHFSCs in the outer root sheath cells in human hair follicles. (**a**) Cav1 immunostaining of normal human scalp hair follicles exhibits colocalization with K15 and CD34, common markers of eHFSC in ORS cells. ORS-Outer Root Sheath. Treatment of normal human scalp hair follicles with Sandalore results in downregulation of Cav1 expression in the bulge at both protein (**b**,**c**) and mRNA levels (**d**). Data are expressed as mean ± SEM, *n* = 13–16 HFs from 2 different donors, Student’s *t*-test, *** *p* < 0.001, GraphPad Prism 6.

Immunohistologically, the bulge area of the human HF displays high mRNA and protein levels of K15 in eHFSCs [41,64], which are relatively quiescent in terms of cell cycle activity [65,66]. Healthy human HFs recruit several mechanisms to protect these eHFSCs from potentially damaging immune responses [67,68]. Central to this is the downregulation of MHC class Ia and II and of the “danger” signal, MHC-class-I-related chain A (MICA), not only in the proximal anagen HF epithelium but also in the bulge, thus restricting antigen presentation and limiting an inflammatory attack on the HF [7,42]. In addition, the human bulge prominently expresses the “no danger signal”, CD200 [42,46]. Furthermore, the bulge shows reduced resident immune cell populations [69] and constitutes a special tissue niche characterized by the expression of potent, locally generated immunosuppressants, including α-melanocyte-stimulating hormone (α-MSH), interleukin 10 (IL-10), insulin-like growth factor 1 (IGF-1) and transforming growth factor β2 (TGF-β2). Combined, these mechanisms restrict immune responses against the bulge and bestow a relative IP on this eHFSC niche (Figure 2) [6,7,42].

Importantly, numerous reports have demonstrated that Cav1 antagonizes guardians of HF-IP (through inhibition of TGF-β and α-MSH signaling) [70,71], and upregulates expression of the key inducer of HF-IP collapse, IFNγ, a key pathogenic cytokine in FFA pathobiology [6,7] (Figure 1b). Selected pro-inflammatory mediators including IFNγ and substance P act as robust upregulators of MHC-Ia and rapidly induce HF-IP collapse [72,73,74,75]. Cav1 has been reported to upregulate expression of substance P and MICA [76,77,78,79], while Cav1 expression itself can be upregulated by TNFα and IL-1β [80,81], whose release is in turn stimulated by Substance P in macrophages and mast cells [82,83], thus facilitating a vicious feedback loop (Figure 1b). Given that these cytokines are non-specifically upregulated in any kind of folliculitis, this could initiate a pathogenesis-promoting vicious circle (Figure 1a). It is important to note that Substance P receptor neurokinin (NK1) localizes to caveolae and that caveolae disruption in either cells (by cyclodextrins) or in Cav1 knockout mice significantly reduces Substance P immunoreactivity and activation of NK1 receptor [84]. Since perifollicular neurogenic inflammation, in which substance P plays a key role [85], has recently been recognized as an additional feature of FFA pathobiology [2], the possibility that Cav1 may facilitate substance P-induced bulge IP collapse deserves careful exploration (Figure 1b). Considering that Substance P and IFN-γ have been previously demonstrated to be upregulators of MHC-I and to rapidly induce HF-IP collapse [73,74], this further substantiates our hypothesis that downregulation of Cav1 may be an alternative approach to inhibit collapse HF immune privilege and thus be a potential avenue for treatment of FFA. It should be noted that these data need to be validated in human hair follicles in order to confirm the physiological relevance of elevated Cav1 expression on IP collapse in scarring alopecia.

**Figure 3:**
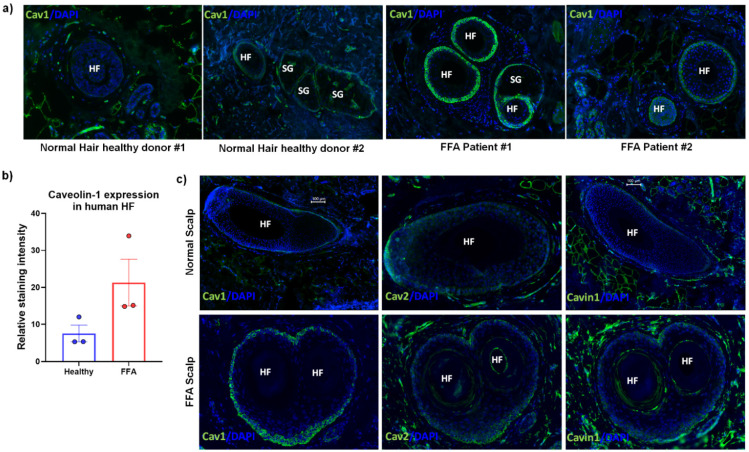
Elevated levels of Cav1 in FFA. (**a**) FFA scalp at the level of the bulge exhibits upregulation of Cav1 expression in comparison to normal scalp from the healthy human donors. HF: Hair follicle; SG: Sebaceous gland. (**b**) Quantification of Cav1 expression from basal layer of outer root sheath cells with error bars corresponding to SEM from *n* = 3 different donors. (**c**) Levels of other structural components of caveolae (Cav2 and Cavin1/PTRF) remain unchanged in comparison to healthy normal scalp, thus indicating a Cav1-specific effect.

Furthermore, not only has expression of Cav1 been demonstrated to be upregulated during EMT [86,87], but Cav1 silencing can also abolish EMT [52,87,88]. Moreover, overexpression of Cav1 can lead to downregulation of E-cadherin and an upregulation of Vimentin [89]. However, it remains unknown whether any of this applies to human HFs and eHFSCs. Yet, the functions of Cav1 in HF and eHFSC biology as well as in HF-IP remain entirely unknown. Preliminary data suggest that Cav1 localizes to the outer root sheath cells of the human HF and co-localizes with K15 and CD34, known markers of eHFSC (Figure 2). Moreover, we have most recently observed abnormal Cav1 protein expression in the bulge region of lesional FFA HFs, where FFA HFs exhibit an elevation of Cav1 expression in the ORS at the level of the HF bulge, in comparison to their healthy scalp counterparts (Figure 3). This effect seems to be Cav1-specific and not as a result of an increased number of caveolae, since levels of other structural components of caveolae (Cav2 and Cavin1/PTRF) remain constant between healthy human scalp and FFA samples (Figure 3c).

**Figure 4:**
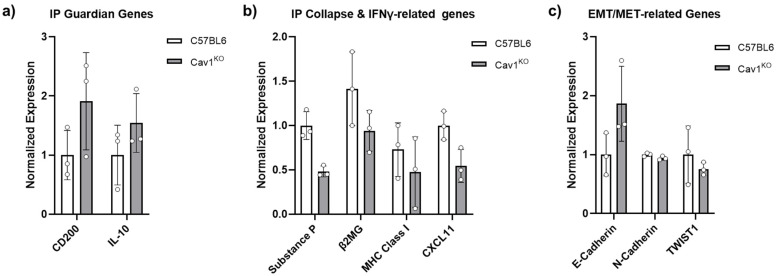
Differential regulation of IP Guardian/Collapse and EMT/MET-related genes in Cav1 knockout mouse skin. (**a**) Full thickness skin biopsy punches from location matched 8-week-old female C57BL6 (i.e., Cav1 WT type) and global Cav1 knockout (Cav1^KO^) mice were utilized to assess expression levels of IP Guardian (CD200, IL-10), IP Collapse (Substance P, β2MG, MHC Class I, CXCL11), and EMT/MET-related genes (E-cadherin, N-cadherin, TWIST1) by qRT-PCR. Cav1^KO^ mouse skin exhibits upregulation of IP guardian genes and a marker of epithelial cells (E-cadherin), as well as a downregulation of IP collapse and EMT related genes. Error bars correspond to standard deviation from *n* = 3 mice.

**Figure 5:**
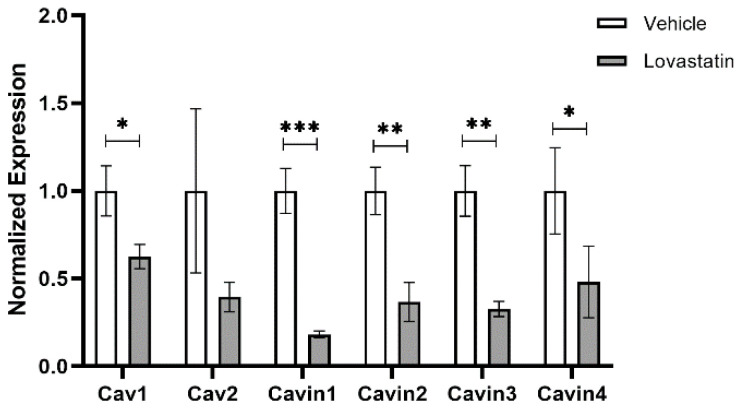
Lovastatin treatment of Outer Root Sheath (ORS) keratinocytes results in downregulation of numerous structural components of caveolae. Primary Outer Root Sheath (ORS) keratinocytes isolated from normal human scalp were treated with 5 µM Lovastatin for 6 h and then utilized to assess expression levels of structural components of caveolae including Cav1, Cav2, Cavin1 (aka PTRF), Cavin2 (aka SDPR), Cavin3 (aka SRBC) and Cavin4 (aka MURC). Cholesterol disruption by Lovastatin resulted in downregulation of each structural component of caveolae. Error bars correspond to SEM from n = 3 technical replicates with statistical significance assessed using two-way ANOVA with Bonferroni correction for multiple comparisons, * *p* < 0.05, ** *p* < 0.01, *** *p* < 0.001.

Interestingly, when we probed expression of key IP collapse/guardian genes in mouse skin isolated from normal (C57BL6) or Cav1 global knockout mice (Cav1^KO^), we observed that Cav1^KO^ mice exhibit an upregulation of IP guardian genes (CD200 and IL-10) and a downregulation of IP collapse and IFNγ-related genes (Substance P, β2MG, MHC Class Ia, CXCL11) (Figure 4). Furthermore, Cav1^KO^ mouse skin exhibited elevated levels of epithelial marker E-cadherin (with no observed changes in mesenchymal marker N-cadherin) and a downregulation of EMT-related gene TWIST1 (Figure 4). The physiological relevance of these data is yet to be determined since they correspond to mouse skin and are not restricted to the hair follicle, and thus need to be validated in human hair follicles. Nonetheless, when we isolated primary human ORS keratinocytes and treated them with lovastatin (5 µM for 6 h), we observed a significant downregulation of Cav1 as well as other structural components of caveolae (Cav2, Cavin-1, -2, -3, -4) (Figure 5), owing to the disruption of cholesterol synthesis by these HMG-CoA reductase inhibitors. However, together the above data and concepts suggest that Cav1 plays a more important regulatory role in several key aspects of human eHFSC physiology and pathology than has so far been appreciated.

## 3. Hypothesis

Specifically, we hypothesize that upregulation of Cav1 as exhibited in human FFA samples (Figure 3) inhibits expression and signaling of IP guardians, while at the same time favoring expression and signaling of IP collapse inducers and EMT, thus ultimately facilitating FFA development. We argue that Cav1 expression may be linked to IP collapse via upregulation of defined molecules that have previously been shown to act as guardians of HF IP (CD200, IL-10, α-MSH, IGF1, TGF-β2), or via downregulation of molecules associated with HF-IP collapse (substance P, MHC class I/II, ß2-microglobulin, IFN-y, CXCL-9, -10, -11, CXCR3 and CD123), or the promotion of EMT in bulge epithelial stem cells (TWIST1, SNAIL, SLUG, vimentin, fibronectin, N-cadherin) [7,8,73,75]. Cav1 has been shown to antagonize the expression of key IP guardian genes (e.g., by inhibiting α-MSH and TGFβ signaling) [70,71], to stimulate expression of key suppressors of IP (substance P and MICA) [76,79], and to promote EMT (vimentin upregulation) [89]. On this basis, we argue that downregulation of Cav1 expression may reverse expression of these key molecules and thus prevent and possibly even reverse IP collapse and EMT, similarly to what our preliminary data comparing skin of Cav1 knockout mice (on a C57BL6 background) to normal C57BL6 mice show (Figure 4). Therefore, topical formulations that target perturbation of Cav1 either by gene knockdown (via nanoparticle siRNA delivery) or pharmacologically (via cyclodextrins or statins) could serve to alleviate development of PLCAs (e.g., FFA) and might complement other therapeutics (e.g., PPARγ modulators).

## 4. How to Probe the Hypothesis?

This hypothesis can be verified by investigating the role of Cav1 in HF IP collapse and EMT induction in the bulge using well-defined in vivo and ex vivo models. These include: (1) an inducible Cav1 knockout mouse model under the control of the K15 promoter to specifically ablate Cav1 in mouse eHFSCs; (2) full-length human HF organ culture in which HF IP collapse and pathological EMT can be experimentally induced in healthy eHFSCs [8,18] in the presence or absence of Cav1-modulatory agents (incl. Cav1 siRNA); (3) organ culture of lesional FFA skin biopsies obtained that demonstrate trichoscopic FFA activity (such as peripilar casts) [7] in the presence or absence of Cav1 inhibitors (e.g., cyclodextrin or Sandalore^®^ (synthetic odorant that we have recently observed to decrease Cav1 expression in the epithelium of healthy human scalp HFs after 6 days of treatment (Figure 2), statins (Figure 5) and siRNA). Using these tractable, clinically relevant and instructive assays, one can investigate changes in eHFSC survival, EMT and IP-collapse markers, and thus probe whether inhibition of Cav1 can indeed prevent/reverse EMT (restore E-cadherin expression, decreased SLUG and vimentin expression) and/or IP-collapse (increased TGF-β1/IGF-1, αMSH and decrease MICA/MHCI expression).

One elementary requirement for validating the above hypothesis obviously is that Cav1 is expressed at all in the bulge region of human scalp HFs, i.e., in the epicenter of FFA pathobiology. Indeed, preliminary data we have just generated suggest that this not only is the case, but that Cav1 is very prominently expressed in the bulge epithelium, where it co-localizes with the key human eHFSC markers [41,66], keratin 15 and CD34 (Figure 2). Given that almost every marker found to be selectively enriched in the bulge of mouse or human HFs has subsequently turned out to be functionally relevant [42,90], chances are that Cav1 is no exception from this rule. Very preliminary evidence that Cav1 protein expression appears to be upregulated in the lesional bulge of FFA patients (Figure 3) further underscores this.

It would also be very good to know whether downregulating Cav1 in human scalp HFs is overall hair growth-inhibitory or, as our hypothesis predicts, hair growth-promoting. In addition, a recent study from Prasad et al. [29], on the impact of Linalool on FFA development, suggested a link between different odorants which might be either pro-FFA (e.g., linalool) or anti-FFA (e.g., Sandalore), possibly due to the regulation of Cav1 in eHFSCs. Previously, we have shown that the synthetic odorant, Sandalore^®^, a selective agonist of olfactory receptor 2AT4, which is expressed also in the bulge, significantly prolongs human hair growth (anagen) by upregulating the intrafollicular production of IGF-1 [18]. Preliminary observations from our lab suggest that Sandalore downregulates Cav1 expression at both the mRNA and protein levels (Figure 2b–d). This pilot observation is in line with the general concept that reducing intrafollicular Cav1 expression/signaling activity may exert hair growth-promoting effects.

Next, the hypothesis can be probed by examining whether downregulation of Cav1 in the HF (by Cav1 siRNA, using our well-established gene silencing protocol in organ-cultured scalp HFs [32,91,92] or pharmacologically by cholesterol disruption of caveolae via cyclodextrins and statins [55,56]) exerts one or more of these effects: (a) strengthening of the bulge IP, as indicated by maximal downregulation of MHC class Ia and ß2-microglobulin expression and upregulation of key IP guardians in the bulge epithelium (e.g., TGF-β1, IGF-1, αMSH); (b) making bulge IP more resistant to IFNγ- or substance P-induced IP collapse, using our corresponding ex vivo assays for testing this [73,74,93]; (c) making it more difficult to experimentally induce EMT (e.g., by increasing E-cadherin and/or decreasing SLUG/vimentin expression via Cav1 inhibition/disruption) in human bulge eSC, using our established ex vivo system [8]; and (d) perhaps even partially restoring human bulge IP and/or inducing MET in human K15 + eHFSCs.

Numerous investigators (including those from our team) have shown that methyl-β-cyclodextrin (MβCD) downregulates Cav1 expression by removing membrane cholesterol and preventing oligomerization of Cav1, the cell membrane [39,40]. Our preliminary data demonstrate that systemic application of Sandalore to human scalp hair follicles similarly downregulates Cav1 expression on both the mRNA and protein levels (Figure 2), and that lovastatin treated primary human outer root sheath keratinocytes also exhibit downregulation of Cav1 expression (Figure 5). Thus, we provide three independent mechanisms for downregulation of Cav1 expression. The hypothesis that Cav1 downregulation by these topically applicable agents may sufficiently attenuate FFA progression/development, thus, is both highly plausible and supported by preliminary evidence, and not only a mere speculation. This plausible, well-supported working hypothesis is also clinically appealing, given the excellent, extensively documented safety profile of cyclodextrins and statins, and the ease at which these drugs could be repurposed for treatment of FFA.

## 5. Translational Significance and Perspectives

The arguments delineated above render it likely that investigating the regulation/modification of Cav1 expression/localization in HFs will identify a powerful novel regulator of human eSC biology in general, and of human eHFSCs in particular. Moreover, the suggested research focus may identify in Cav1 a promising novel target for therapeutic intervention—not only in FFA management, but also for other forms of cicatricial alopecia [94], as well as for other stem cell- and IP-related diseases such as multiple sclerosis [95] and autoimmune uveitis [96,97] and diseases characterized by pathological EMT like scleroderma [98]. We have previously shown that Sandalore selectively activates OR2AT4 in HFs and that expression of Cav1 mRNA and protein levels is decreased after Sandalore treatment for 6 h and 6 days, respectively [32]. This suggests that the specific activation of this OR is directly linked to Cav1 expression in human scalp HFs. However, to functionally confirm this link, it will be necessary to perform OR2AT4 silencing in the presence of Sandalore. Although it is plausible to assume that Sandalore (as a OR2AT4 agonist) may induce localization of the odorant receptor to caveolae (similar to a number of other G-protein coupled receptors (as well as G-proteins) that have been identified over the past 20 years [99,100,101,102,103], this has yet to be experimentally verified and is something that we are actively pursuing.

There is, however, a striking resemblance between topical Sandalore treatment and Cav1 depletion in skin including increased cAMP activity and subsequent Ca^2+^ increase [104], activation of EGFR and subsequent MAPK signaling, as well as increased rates of proliferation, migration and epidermal re-epithelialization [55,56,105]. Thus, this link may not only be functionally relevant to hair follicle physiology, but also wound re-epithelialization (which is promoted by OR2AT4 activation by Sandalore [105]) and possibly other cutaneous functions. To the best of our knowledge, the data presented in the current manuscript provide the first evidence for a therapeutically targetable linkage between olfactory receptors (namely OR2AT4) and Cav1 (by Sandalore) in any human disease context.

Since statins have been utilized extensively for a number of diseases (skin and others) over the past few decades, the ease at which they could be repurposed for treatment of FFA, and possibly other forms of scarring alopecia, makes them an attractive option. It should be noted, however, that sustained topical usage of statins may lead to aberrant sebaceous gland activity and sebum composition. Unfortunately, our current understanding regarding the role statins may have in sebaceous gland activity is limited and based on mouse models. For example, Zhang et al. [106] demonstrated that excess cholesterol production in *Gk-5*-deficient mice (model of alopecia) disrupts hair follicle development and cycling in these mice, which can be partially rescued by localized simvastatin treatment. Cakmak et al. [107] demonstrated that diabetic mice (STZ-induced) exhibit altered lipid synthesis in their sebaceous glands, which can be partially reversed by intraperitoneal simvastatin treatment; however, they exhibited no difference in epidermal/dermal thickness, proliferative activity or dermal composition [107,108]. Since HMG-CoA reductase is found active in isolated human apocrine glands, sebaceous glands and hair follicles, it is possible that sustained topical statin treatment may reduce HMG-CoA activity and thus result in lower cholesterol production and amount to a decline in sebaceous function and dry skin. However, these results are yet to be recapitulated in human specimens.

Probing our hypothesis on the role of Cav1 in the development of FFA and other PLCAs and the importance of downregulating it to strengthen and/or restore IP in the bulge of human HFs (Figure 1) may not only introduce a new pathomechanism, but could also invite a promising and innovative treatment strategy for these debilitating, irreversible hair loss disorders. If we can confirm that Cav1 expression is indeed regulated by some odorants, the development of cosmeceutical instead of costly drugs, which must overcome much higher regulatory hurdles, would greatly facilitate patient access to therapy.

## Data Availability

The data presented in this study are available from the corresponding author upon request.
